# Early Vascular and Functional Changes after Vitreoretinal Surgery: A Comparison between the Macular Hole and Epiretinal Membrane

**DOI:** 10.3390/diagnostics11061031

**Published:** 2021-06-03

**Authors:** Rossella D’Aloisio, Paolo Carpineto, Agbéanda Aharrh-Gnama, Carla Iafigliola, Luca Cerino, Marta Di Nicola, Annamaria Porreca, Lisa Toto, Rodolfo Mastropasqua

**Affiliations:** 1 Ophthalmology Clinic, Department of Medicine and Science of Ageing, University “G. d’Annunzio” Chieti-Pescara, via dei Vestini 31, 66100 Chieti, Italy; p.carpineto@gmail.com (P.C.); gnamaomer@tiscali.it (A.A.-G.); carlaiafigliola@hotmail.it (C.I.); lucacerino92@gmail.com (L.C.); l.toto@unich.it (L.T.); 2 Laboratory of Biostatistics, Department of Medical, Oral and Biotechnological Sciences, University “G. d’Annunzio” Chieti-Pescara, via dei Vestini 31, 66100 Chieti, Italy; mdinicola@unich.it; 3 Department of Economic Studies, University “G. d’Annunzio” Chieti-Pescara, Viale Pindaro, 65100 Pescara, Italy; porreca.annamaria@unich.it; 4 Institute of Ophthalmology, University of Modena and Reggio Emilia, 41121 Modena, Italy; rodolfo.mastropasqua@gmail.com

**Keywords:** widefield swept-source optical coherence tomography angiography, vitreoretinal surgery, macular hole, epiretinal membrane

## Abstract

(1) Background: The aim of this observational comparative study was to investigate early retinal vascular and functional changes in patients undergoing vitreoretinal surgery for idiopathic epiretinal membrane (iERM) or macular hole (MH) using a widefield swept-source optical coherence tomography angiography (WSS-OCTA). (2) Methods: Forty one diseased eyes were enrolled in the study. Twenty three eyes with iERM diagnosis (ERM group) underwent 25-gauge vitrectomy with inner limiting membrane (ILM) and MER peeling, while eighteen eyes with MH (MH group) underwent 25-gauge vitrectomy with inverted flap technique. Functional and anatomical/perfusion parameters were evaluated pre- and postoperatively in all eyes by means of WSS-OCTA system, microperimetry (MP3), best corrected visual acuity assessment, central macular thickness (CMT) and MH diameter calculation. For each eye, 12 × 12 mm OCTA volume scans were acquired by a retinal specialist and a semi-automated algorithm was used for a quantitative vessel analysis of the superficial capillary plexus (SCP), deep capillary plexus (DCP) and choriocapillaris (CC). In detail, perfusion density (PD) of the SCP, DCP and CC was evaluated in four circles (one central in the macular area of 5 mm diameter; three midperiphery circles (temporal, superior and inferior) of 3 mm). In addition, the vessel length density (VLD) of the SCP and DCP for the same circles was quantified. (3) Results: In the MH group, PD of the SCP significantly increased in the macular area (*p* = 0.018) and in the superior ring (*p* = 0.016); PD of the DCP significantly increased in the macular area (*p* = 0.015) and in the superior and inferior ring (*p* = 0.016) 3 months after surgery. In the ERM group, PD of the SCP and DCP significantly increased in the macular area and superior ring, respectively (*p* = 0.001; *p* = 0.032), 3 months after surgery. During follow-up there was a significant improvement in terms of functional (Best corrected visual acuity, *p* = 0.007 and *p* = 0.029; microperimetry ((MP3) 10°, *p* = 0.003 and *p* = 0.004; MP3 2°, *p* = 0.028 and *p* = 0.003 in MH group and ERM group respectively) and anatomical parameters (CMT, *p* = 0.049 in ERM group; hole complete closure in MH group). (4) Conclusions: After vitreoretinal surgery, early retinal vascular and functional changes can be promptly observed and quantified to monitor and potentially predict surgery outcomes. Widefield OCTA devices allow for a detailed microvasculature analysis of retina and choriocapillaris in the macular area and in the periphery, showing a different behaviour of retinal sectors in two distinct vitreoretinal disorders.

## 1. Introduction

The trend of performing macular surgery for either macular hole (MH) or epiretinal membrane (ERM) has remarkably increased by 7.8% from 2010 to 2017 [1].

Although many causes for MH onset have been hypothesized such as high myopia, inflammation conditions and trauma, idiopathic causes are the most frequent with a prevalence of 1 in 3300 in the over 60 age population [2,3,4].

Epiretinal membrane can be idiopathic or secondary to diabetes, retinal laser treatment or inflammatory conditions [2,3,4].

Pars plana vitrectomy with inner limiting membrane (ILM) peeling combined with gas tamponade and ERM peeling is considered the first line surgical approach for the treatment of MH and ERM, respectively [2,5].

Usually, more than 90% of idiopathic MHs appear to be completely closed after surgery [2,5,6].

An effective MH closure with a parallel functional recovery depends on progressive inner retina restoration followed by the outer retina layers, while a complete ERM and ILM peeling is essential to avoid its recurrence [2,7].

Some studies have focused on possible prognostic factors for a successful long-term surgery of these two vitreoretinal conditions. The introduction of OCTA in the clinical setting has allowed a detailed evaluation of retinal vasculature status of all layers pre and post surgery [8,9,10]. Widefield SS OCTA has given the chance to detect, simultaneously in a single scan, the centre and periphery of the retinal and choroidal perfusion.

Widefield devices are able to visualize in vivo retinal microarchitecture changes of eyes with vitreoretinal diseases and after surgery.

Previous studies have focused their attention on retinal vasculature features as possible predictive factors for surgical success, describing that vitreoretinal tractions may lead to retinal perfusion changes in a reversible fashion, likely due to a direct mechanical effect of vitreous traction on retinal vessels [9].

The aim of this study was to provide a quantitative and qualitative mapping and analysis of early retinal and choriocapillaris (CC) perfusion changes after vitreoretinal surgery using an advanced widefield SS OCTA system. A comparison between the two different macular diseases was assessed in terms of perfusion/anatomical and functional parameters of different retinal sectors.

## 2. Materials and Methods

### 2.1. The Materials and Study Participants

In this observational cross-sectional study, a total of 41 eyes were recruited after approved informed consent was obtained from all participants.

The study was approved by our Institutional Review Board (Department of Medicine and Science of Ageing, University G. d’Annunzio Chieti-Pescara, Chieti, Italy) on the 6th of October 2020 (FE 20) and adhered to the tenets of the Declaration of Helsinki. A total of 41 diseased eyes were enrolled: 23 eyes with idiopathic ERM diagnosis (ERM group; 43.5% females, 56.5% males; mean age of 63.1 years (57.2–64.8)) underwent 25-gauge vitrectomy with ILM and ERM peeling, 18 eyes with idiopathic MH (MH group; 44.4% females; 55.6% males; mean age of 63.0 years (55.5–63.9)) underwent 25-gauge vitrectomy with inverted flap technique.

All subjects were imaged with widefield swept-source PLEX Elite 9000 device (Carl Zeiss Meditec Inc., Dublin, CA, USA). Moreover, all patients received a complete ophthalmologic evaluation, including best corrected visual acuity (BCVA) assessment, intraocular pressure (IOP), minimal and basal diameters of MH and central macular thickness (CMT) pre- and postoperatively, with a 3-month follow-up.

Macular sensitivity was detected using MP-3 (Microperimetry-3, Nidek, Japan).

Inclusion criteria were: (1) diagnosis of iERM (3 stage, according to Govetto classification system) [11] or MH diagnosis (4 stage according to Gass classification system), (2) no history of previous ocular surgery, (3) iERM duration ≤6 years or MH duration ≤6 months, (4) bulbar axial length of no more than 25 mm.

Exclusion criteria were: (1) evidence or history of ocular conditions such as retinal detachment, retinal vascular occlusions, uveitis, high myopia, trauma; (2) evidence or history of systemic disorders, including diabetes and systemic hypertension; (3) poor image quality or no patient collaboration.

### 2.2. Perfusion Parameters

#### 2.2.1. Image Acquisition

Subjects underwent OCTA imaging using the PLEX Elite 9000 device (Carl Zeiss Meditec Inc., Dublin, CA, USA) which uses a swept laser source with a central wavelength of 1050 nm (1000–1100 nm full bandwidth) and operates at 100,000 A-scans per second. 

For each eye, three 12 × 12 mm OCTA volume scans were acquired preoperatively, at 1 and 3 month after surgery. FastTrac motion correction software was used while the images were acquired. Poor quality images (signal strength index <8) with either significant motion artifact or incorrect segmentation were excluded. 

All selected images were carefully visualized by a retinal specialist to ascertain the correctness of segmentation and in case of erroneous recognition by the software of the position of the boundaries of the ILM and retinal pigment epithelium, manual correction was performed using the segmentation and propagation editing tool from the device.

#### 2.2.2. Image Processing

The main outcome measures were: (1) superficial capillary plexus (SCP) perfusion density (PD); (2) SCP vessel length density (VLD); (3) deep capillary plexus (DCP) PD; (4) DCP VLD; (5) CC PD.

In order to quantify these variables, a slightly modified previously reported semi-automated algorithm was employed [10,12]. In brief, for each eye, en face OCTA images segmented at the SCP, DCP and CC level were imported into ImageJ software version 1.50 (National Institutes of Health, Bethesda, MD, USA; available at http://rsb.info.nih.gov/ij/index.html, last accessed on 17 April 2021) and, consequently were processed after binarization were skeletonized, as previously described [10].

**Figure 1:**
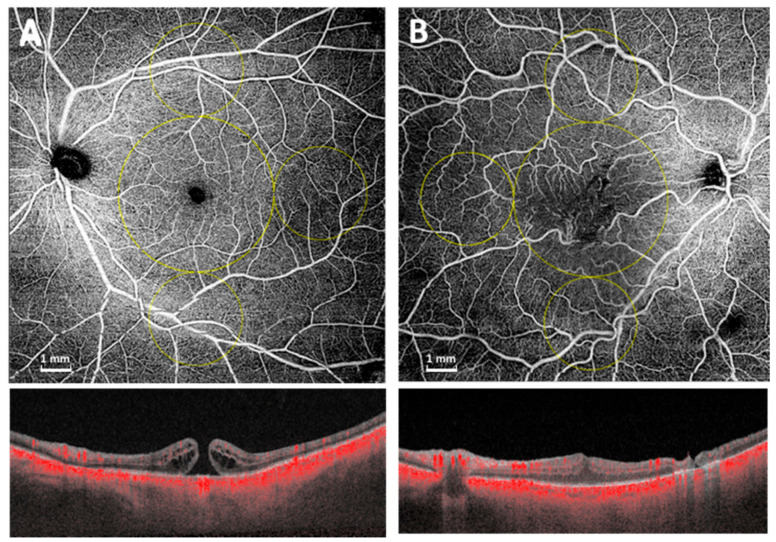
Widefield OCTA scan (12 × 12 mm) of superficial capillary plexus and structural OCT of macular hole (**A**) and epiretinal membrane (**B**). The quantitative analysis was performed in the macular region, which was defined as a circular annulus around the fovea with diameter of 5 mm, excluding the foveal avascular zone and in the midperiphery 3 circles (temporal, superior and inferior of 3 mm).

The quantitative analysis was thus performed in the macular region, which was defined as a circular annulus around the fovea with diameter of 5 mm and excluding the foveal avascular zone and in the midperiphery 3 circles (temporal, superior and inferior of 3 mm) as previously described [13] and as shown in Figure 1.

### 2.3. Functional Parameters

#### Microperimetry

Microperimetry was performed by means of MP-3.

All patients were dilated with tropicamide 1% eye drops and, after pre-test training, 5 min were allotted for adaptation to the dark. An automated eye tracking system providing real-time compensation for eye movements and improving presentation of a stimulus at a predefined retinal location was used. During the test, the patient was encouraged to fixate on a red cross target, 2° in diameter, on a white monochromatic background at 4/31.4 asb. Then, retinal sensitivity was tested using a customized radial grid centred on the fovea with 29 Goldman III stimuli covering the central 10° and 2°. The stimulus intensity ranged from 0 dB to 20 dB (0 dB corresponded to the strongest signal intensity of 127 cd/m^2^) in 1-dB steps, and the duration of each stimulus was 200 milliseconds. To assess central macular retinal sensitivity, differential light threshold values were compared by calculating the mean of the 2° and 10° of the macular area, which was averaged automatically by the MP-3 software programme for the mean sensitivity in a polygon.

To assess fixation, fundus movements were tracked during the examination, and where the patient gazed on the fixation target the fixation pattern was assessed. To evaluate fixation location, the fixation target centred on the fovea was defined. Fixation location was expressed as a percentage of fixation points located within the 2° and 4° central areas. Eyes with >50% of the preferred fixation points located within the central area were classified as having predominantly central fixation. Eyes with >25% but <50% of the preferred fixation points located within the central area were classified as having poor central fixation. Eyes with <25% of the preferred fixation points located within the central area were classified as having predominantly eccentric fixation.

### 2.4. Surgical Procedure

ERM group underwent 25G 3-port pars plana vitrectomy with ERM and ILM peeling after ERM and ILM staining with a combination of 0.15% trypan blue, 0.025% brilliant blue G, and 4.00% polyethylene glycol (MembraneBlue-DualTM, DORC International, Zuidland, The Netherlands). MH group underwent 25G 3-port pars plana vitrectomy with ILM peeling with inverted flap technique and SF6 gas tamponade after ILM staining with MembraneBlue-DualTM. ILM peeling was performed symmetrically around the fovea.

In MH group after surgical procedure a facedown position was maintained for 7 days.

All procedures were performed by a single, experienced surgeon (R.M.).

In total, 8 eyes were phakic and 33 were pseudophakic. In phakic eyes, combined phacovitrectomy with intraocular lens implantation in the capsular bag was performed. 

Intraoperative OCT was used to check macular hole closure and a complete ERM peeling. 

No intra- and postoperative complications were reported.

### 2.5. Statistical Analysis

The Shapiro–Wilk test was performed to evaluate the normal distribution of the data. Median, 1st and 3rd quartile were reported as summary and variability measures, respectively. The Mann–Whitney U test was used to assess differences between groups. The Friedman test was used to evaluate differences over time within experimental groups (MH and ERM). Bonferroni correction was used for pairwise correction. As the Friedman test does not handle missing data, missing values were treated using the Skillings–Mack statistic test [14]. This nonparametric statistical test is helpful for the data obtained from block designs with missing observations occurring randomly. The Skillings–Mack test p-value could be obtained with the Monte Carlo method. The Monte Carlo method is recommended for approximating p-values when many ties and/or small designs with missing values are conducted. Spearman’s Rho correlation coefficient was applied to evaluate the correlation among functional and retinal perfusion variables. Statistical analysis was performed using R software environment for statistical computing and graphics version 3.5.2 (R Foundation for Statistical Computing, Vienna, Austria; https://www.R-project.org/, last accessed on the 17 April 2021).

## 3. Results

A total of 41 eyes were enrolled and considered in the statistical analysis.

**Figure 2:**
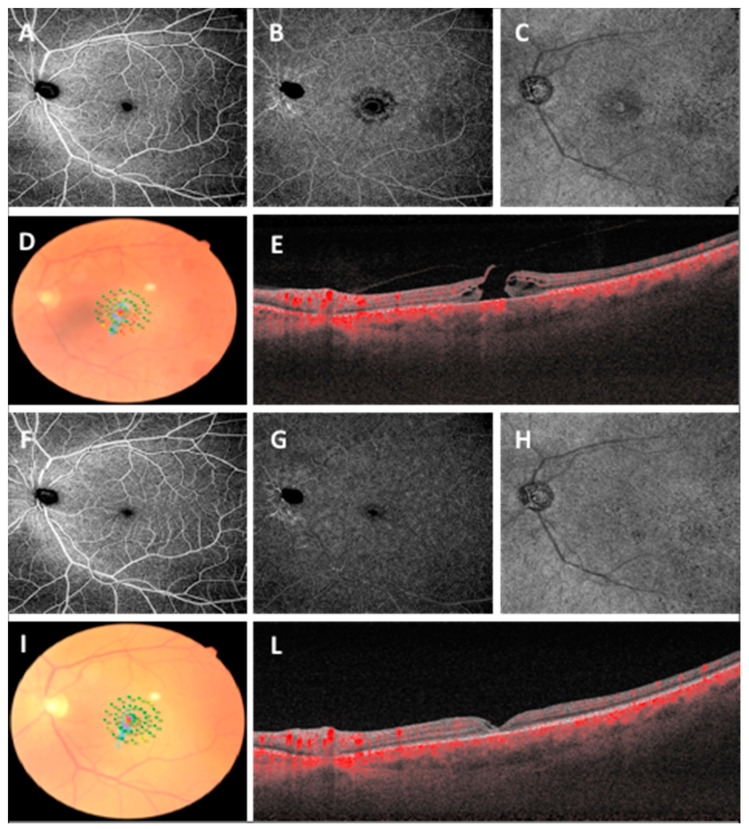
Widefield OCTA scans (12 × 12 mm) of superficial capillary plexus (**A**), deep capillary plexus (**B**), choriocapillaris (**C**), microperimetry (**D**) and OCT scan (**E**) before vitreoretinal surgery in macular hole. Widefield OCTA scans (12 × 12 mm) of superficial capillary plexus (**F**), deep capillary plexus (**G**), choriocapillaris (**H**), microperimetry (**I**) and OCT scan (**L**) at 3-month follow-up.

**Figure 3:**
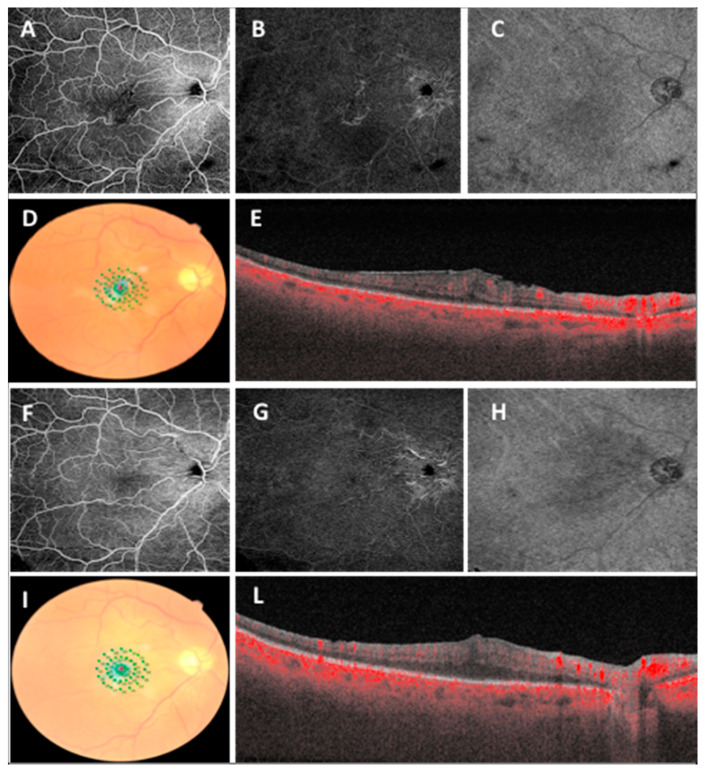
Widefield OCTA scans (12 × 12 mm) of superficial capillary plexus (**A**), deep capillary plexus (**B**), choriocapillaris (**C**), microperimetry (**D**) and OCT scan (**E**) before vitreoretinal surgery in epiretinal membrane. Widefield OCTA scans (12 × 12 mm) of superficial capillary plexus (**F**), deep capillary plexus (**G**), choriocapillaris (**H**), microperimetry (**I**) and OCT scan (**L**) at 3-month follow-up.

No differences were found in terms of age or gender in the two groups (MH and ERM groups; *p* > 0.05). After surgery, during whole follow-up, a statistically significant improvement was observed in terms of functional (BCVA, *p* = 0.007 and *p* = 0.029; MP3 10°, *p* = 0.003 and *p* = 0.004; MP3 2°, *p* = 0.028 and *p* = 0.003 in the MH group and the ERM group, respectively) and anatomical parameters (CMT, *p* = 0.049 in the ERM group; hole complete closure in the MH group) (Table 1, Figure 2 and Figure 3).

In the MH group, PD of the SCP significantly increased in the macular area (*p* = 0.018) and in the superior ring (*p* = 0.016); PD of the DCP significantly increased in the macular area (*p* = 0.015) and in the superior and inferior ring (*p* = 0.017; *p* = 0.016) at 3-month follow-up after surgery (Table 2). In the ERM group, PD of the SCP and DCP significantly increased in the macular area and superior ring, respectively (*p* = 0.001; *p* = 0.032; Table 2) 3 months after vitreoretinal surgery.

At the 3-month follow-up, PD of the temporal sector was the lowest in both groups, except for the DCP of the ERM group.

In the MH group, the SCP VLD increased significantly in all retinal sectors except for the superior ring if compared with baseline values; while the DCP VLD increased significantly only in the macular area (*p* = 0.016; Table 3).

Conversely, in the ERM group, VLD did not change significantly during the follow-up.

No differences were found in terms of PD and VLD of the CC for the whole 3-month follow-up in both groups.

A significant positive correlation was found in the MH group between perfusion parameters of both central and peripheral retina and macular sensitivity. Indeed, a statistically significant positive correlation was found in the ERM group between macular PD and macular sensitivity. Simultaneously, in the retinal periphery, only the DCP PD was significantly associated with microperimetry (Table 4).

In the MH group, BCVA showed a significant negative correlation with macular SCP PD, macular SCP VLD and peripheral DCP VLD (Table 4).

## 4. Discussion

Over the years, advances in ocular imaging technology have allowed for a more detailed evaluation of morphological and functional retinal features after MH or ERM surgery [15,16,17].

With the widespread use of OCTA in clinical practice, there is great interest in the perfusion parameters of retina and choroid and the changes after vitreoretinal surgery, to better understand some predictive factors of successful postoperative outcomes [8,9,10,15,16,17,18,19].

Due to high resolution and fast image acquisition time, SS OCTA models are widely used in the detection of vascular changes in various retinal diseases [15,16,17,18,19,20]. In our work, we aimed at investigating functional and anatomical features of macular area and midperiphery of the retina and choriocapillaris in patients before and after vitreoretinal surgery for MH and ERM using a widefield SS OCTA device. The unique angle of this work is that it compares two different vitreoretinal diseases describing early perfusion changes of both the central area and midperiphery sectors of the retina after the surgical procedure.

Modifications in the vascular microarchitecture of both the SCP and DCP were previously described in these two vitreoretinal diseases, likely due to mechanical displacement of retinal vessels [16,21]. Tractions caused by ERM lead to a vessel displacement due to anteroposterior and centripetal forces with consequent macular distortion and a reorganization of inner retinal layers. In MH, a mechanical retinal stress, caused by vitreal tractions, induces changes in retinal structure and circulation [22]. Kumagai et al. [22] reported a centripetal shift of foveal vascular network after vitrectomy and ILM peeling.

Our findings revealed a significant increase in macular perfusion in both groups during a 3-month follow-up after vitrectomy. In detail, a macular PD rise of both capillary plexuses was observed in the MH group and only at the SCP level in the ERM group.

After surgery repairing the macular hole, the foveolar area returned approximately to its original position with the recovery of visual function. Interestingly, at 30 days after surgery, a reduction in macular PD of the DCP was observed with a successive significant rise at 3-month follow-up, likely due to slow reabsorption of the intraretinal cysts wall typically localized at the edge of the hole between the inner nuclear layer and outer plexiform layer where the capillary bed of the DCP is. 

In the ERM cohort of patients, overall perfusion density of both capillary plexuses in the retinal periphery did not change significantly during follow-up, probably because some tractional forces may persist despite their release after surgery. Besides, it can be hypothesized that the preoperative capillary sub-occlusion could take a longer time for microvessel network recanalization in the midperiphery of retina. 

As known, macular hole and epiretinal membrane are typically caused by the tangential traction of the cortical vitreous [23,24,25]. In these two pathological conditions, a morphologic remodeling of the retina and choroid in the macular area has previously been described [8,23,26].

Subjects with ERM usually have a smaller FAZ than healthy eyes [17]. Mao et al. found no difference in foveal vessel density between eyes with iERM and fellow healthy eyes or between pre- and post-operative findings [17]. However, there were several limitations in their study, as microperimetry was not analyzed at all.

On the contrary, our cohort of patients who underwent surgery showed a significant improvement in terms of functional parameters (visual acuity and macular sensitivity) during whole post surgical follow-up.

Regarding perfusion findings, our results confirmed early changes in the morphology and topography of retinal vasculature after surgery, similarly to previous works. 

Indeed, Bacherini et al. [18] have described a significant rise in macular perfusion (3 × 3 scan) after a 6-month follow-up after surgery in subjects with ERM. 

However, to the best of our knowledge, no studies have provided precise data on early retinal flow modifications using a WSS OCTA system, comparing simultaneously the two pathologies that interestingly showed different behavior, and reporting both periphery and macular sectors in the same analysis. 

In the MH group, VLD increased significantly both in the macular area and in the periphery after surgery, while in the ERM group VLD did not change significantly during the follow-up, thus suggesting a different recovery time for postoperative vessel structure restoration.

It is also true that ERM with a rich intraretinal fluid component may weaken the reflected OCTA signal intensity from deeper layers; nevertheless, we included a similar duration of ERM diagnosis and at the same stage to avoid bias related to different disease severity. Moreover, as large blood vessels of the SCP may affect deeper layers, projections removal software was used for all scans, therefore potential measurement errors were universally applied to all subjects.

It is known that ILM peeling can lead to a reduction in temporal retinal regions thickness with foveal displacement nasally [27]. Moreover, after vitreoretinal surgery with ILM peeling a nasal macular dragging has been previously described with the consequent shift of temporal vessels towards the optic disc [28]. Indeed, in our work PD of the temporal sector was the lowest in both groups, except for the DCP of the ERM group, at the 3-month follow-up after surgery.

Many risk factors for vitreoretinal diseases onset have been described over the years [29,30]. A thin choroid and a reduction of foveolar choroidal blood flow seem to play an important role in MH onset [31]. A prospective study of 25 patients with unilateral IMH who underwent vitrectomy reported that the choriocapillary flow area and parafovea vessel density significantly increased at one month after vitrectomy, showing the reversible behavior of CC macular circulation postoperatively [8]. Moreover, choriocapillaris vasculature was negatively correlated with preoperative macular hole diameters, although was independent from baseline visual acuity. Conversely, our data showed no significant changes of CC in both diseases after vitrectomy, in the macular area and in the midperiphery. It is still controversial as to whether choroidal thickness alteration is a leading factor in macular hole onset due to the challenging analysis of choroidal circulation, despite high tech devices [32,33].

In both vitreoretinal groups, a positive significant correlation was found between perfusion parameters of both the central and peripheral retina and macular sensitivity. 

Moreover, the MH group showed a negative significant correlation between perfusion parameters and BCVA. 

The quantification and mapping of retinal microcirculation and its relationship with functional data may become an easy and early prognostic tool of the final outcome after vitreoretinal surgery. This study is of great interest for understanding the underlying mechanisms in perfusion changes after surgery and potentially discovering reliable biomarkers and possible novel therapeutic strategies. The strength of this work is the simultaneous comparison of both perfusion/anatomical and functional features of two distinct vitreoretinal pathologies, and the retinal vasculature assessment of both periphery and macular sectors in the same analysis by means of a state-of-the-art widefield OCTA platform. 

One of the main limitations of the study was that structural OCT features were not discussed in our study given its focus on the use of widefield OCTA; they can be included in future studies with a longer follow-up.

## 5. Conclusions

In conclusion, our study revealed that after vitreoretinal surgery early retinal vascular and functional changes can be promptly observed and easily quantified to monitor and potentially predict surgery outcomes. Widefield OCTA devices allow for a detailed microvasculature analysis of the retina and choroid in the macular area and in the periphery, showing the different behavior of two distinct vitreoretinal disorders. Retinal microvasculature as well as retinal tissues are involved and distorted by vitreal traction forces; nevertheless, these changes seem to be differently impaired in ERM and MH disorders.

## Figures and Tables

**Table 1:** Median and quartiles (Q1; Q3) for MH (macular hole) and ERM (epiretinal membrane) reported for baseline and up to 90 days, respectively, for every variable in the study.

Variable	MH			
	Baseline	30 Days	90 Days	*p*-Value
MP310° db	16.5 (12.2;20.1)	23.4 (16.0;24.3)	27.0 (26.2;27.5) ^(b)(t)^	0.003
MP3 2° db	15.2 (5.50;15.4)	19.5 (15.8;21.0)	24.2 (24.2;24.2) ^(b)(t)^	0.028
BCVA	1.00 (0.50;1.00)	0.35 (0.18;0.55) ^(b)^	0.40 (0.30;0.40) ^(b)(t)^	0.007
CMT	\	\	\	\
	**ERM**			
	**Baseline**	**30 Days**	**90 Days**	***p*-Value**
MP310° db	22.0 (18.4;24.0)	24.9 (21.9;25.5)	26.9 (26.5;27.2) ^(b)(t)^	0.004
MP3 2° db	21.0 (17.0;22.8)	23.2 (21.8;24.2)	27.0 (26.6;27.4) ^(b)(t)^	0.003
BCVA	0.50 (0.40;0.80)	0.40 (0.20;0.60)	0.20 (0.10;0.6) ^(b)(t)^	0.029
CMT	472.0 (430.0;553.0)	349.0 (312.0;396.0) ^(b)^	290.0 (288.0;399.0) ^(b)(t)^	0.049

The Friedman test *p*-value is shown. (b) = the median at the time points of evaluation for experimental group’s results statistically different from the baseline value. (t) = the median at the time points of evaluation for experimental group’s results statistically different from 30 days’ time point.

**Table 2:** Median and quartiles (Q1; Q3) for MH (macular hole) and ERM (epiretinal membrane) reported for baseline and up to 90 days, respectively, for every variable in the study.

PD				
	MH			
SCP	Baseline	30 Days	90 Days	*p*-Value
Macular ring	37.5 (35.6;39.5)	43.9 (31.3;51.3) ^(b)^	45.5 (39.2;51.8) ^(b)(t)^	0.018
Superior ring	42.0 (34.2;50.5)	47.0 (31.7;62.5) ^(b)^	51.8 (36.0;67.5) ^(b)(t)^	0.016
Temporal ring	18.2 (18.3;18.8)	26.5 (17.8;35.2)	25.7 (12.6;38.7)	0.955
Inferior ring	44.9 (41.9;48.1)	49.4 (43.1;54.9)	49.1 (40.1;58.2)	0.422
**DCP**				
Macular ring	45.5 (39.1;52.1)	40.1 (32.9;47.3) ^(b)^	49.2 (48.5;49.8) ^(t)^	0.015
Superior ring	46.1 (43.1;51.1)	38.8 (25.1;51.8) ^(b)^	50.1 (40.4;59.8) ^(t)^	0.017
Temporal ring	35.5 (29.8;40.7)	32.5 (23.1;42.2)	33.6 (25.3;41.9)	0.999
Inferior ring	48.4 (37.9;58.5)	38.0 (31.8;44.8) ^(b)^	50.3 (45.5;55.0)^(t)^	0.016
**ERM**				
**SCP**	**Baseline**	**30 Days**	**90 Days**	***p*-Value**
Macular ring	29.9 (23.0;35.5)	22.2 (18.2;27.8) ^(b)^	36.3 (27.9;41.5) ^(t)^	0.001
Superior ring	37.0 (28.7;42.1)	37.2 (29.4;39.2)	39.6 (32.3;42.2)	0.514
Temporal ring	25.6 (18.8;28.6)	26.3 (19.3;34.5)	26.2 (19.3;34.3)	0.662
Inferior ring	32.9 (23.5;38.3)	35.0 (31.4;40.0)	35.0 (32.4;39.0)	0.838
**DCP**				
Macular ring	32.9 (22.5;36.1)	30.1 (29.4;34.3)	30.6 (29.7;31.9)	0.943
Superior ring	25.8 (24.6;33.7)	20.5 (19.5;25.8) ^(b)^	23.0 (18.9;33.4) ^(b)(t)^	0.032
Temporal ring	29.2 (22.7;40.7)	27.0 (18.0;30.9)	26.9 (15.3;30.4)	0.701
Inferior ring	25.4 (21.9;34.1)	26.7 (19.8;29.1)	27.8 (24.4;29.1)	0.666

The Friedman test *p*-value is shown. PD: perfusion density. (b) = the median at the time points of evaluation for experimental group’s results statistically different from baseline value. (t) = the median at the time points of evaluation for experimental group’s results statistically different from 30 days′ time point.

**Table 3:** Median and quartiles (Q1; Q3) for MH (macular hole) and ERM (epiretinal membrane) reported for baseline and up to 90 days, respectively, for every variable in the study.

VLD				
	MH			
SCP	Baseline	30 Days	90 Days	*p*-Value
Macular ring	13.9 (12.4;14.8)	16.7 (13.4;18.5) ^(b)^	15.6 (14.4;16.7) ^(b)^	0.016
Superior ring	14.5 (13.0;18.4)	15.0 (12.0;20.2)	16.2 (13.3;19.1)	0.423
Temporal ring	7.2 (7.92;13.7)	10.6 (10.7;13.5) ^(b)^	9.20 (5.38;13.0) ^(t)^	0.015
Inferior ring	15.2 (14.5;16.6)	16.1 (11.9;18.0) ^(b)^	15.6 (14.0;17.2) ^(t)^	0.015
**DCP**				
Macular ring	17.1 (14.4;20.8)	16.1 (13.6;18.1) ^(b)^	17.4 (16.5;18.4) ^(t)^	0.016
Superior ring	15.6 (13.8;18.9)	16.6 (14.8;19.5)	17.1 (15.9;18.4)	0.420
Temporal ring	15.8 (12.9;16.8)	12.2 (12.0;16.1)	12.4 (10.7;14.2)	0.422
Inferior ring	16.7 (14.7;20.6)	13.3 (10.9;17.1)	16.7 (16.3;17.1)	0.421
**ERM**				
**SCP**	**Baseline**	**30 Days**	**90 Days**	***p*-Value**
Macular ring	10.2 (8.01;12.7)	9.83 (8.13;11.6)	10.9 (9.90;12.6)	0.465
Superior ring	11.2 (9.44;13.5)	11.8 (9.73;13.0)	13.9 (10.4;14.2)	0.465
Temporal ring	8.73 (7.12;9.70)	10.3 (8.66;11.4)	10.1 (7.95;12.6)	0.161
Inferior ring	8.29 (7.61;11.8)	9.64 (8.67;12.8)	10.1 (8.76;13.0)	0.840
**DCP**				
Macular ring	14.0 (10.9;15.2)	12.3 (11.3;12.8)	12.2 (11.4;12.3)	0.662
Superior ring	11.5 (9.92;14.7)	10.9 (9.30;13.3)	9.47 (8.54;9.61)	0.055
Temporal ring	11.6 (9.86;16.5)	11.8 (9.02;13.1)	12.2 (6.28;12.6)	0.161
Inferior ring	12.0 (10.5;13.9)	11.0 (9.80;13.2)	11.0 (11.0;13.0)	0.701

The Friedman test *p*-value is shown. VLD: Vessel Density. (b) = the median at the time points of evaluation for experimental group’s results statistically different from baseline value. (t) = the median at the time points of evaluation for experimental group’s results statistically different from 30 days’ time point.

**Table 4:** Spearman’s Rho correlation coefficient among absolute variation of functional parameters and absolute variation of retinal perfusion variables.

	MH			ERM		
	MP 10°	MP 2°	BCVA	MP 10°	MP 2°	BCVA
**PD**						
**SCP Macular**	0.980 **	0.965 **	−0.985 **	0.800 *	0.800 *	−0.600
**DCP Macular**	0.600	0.600	0.200	−0.098	0.515 *	−0.387
**SCP Peripheral**	0.910 **	0.975 **	0.257	−0.064	−0.182	−0.361
**DCP Peripheral**	0.435 *	0.450 *	0.089	0.494 *	−0.418	−0.182
**VLD**						
**SCP Macular**	0.910 **	0.975 **	−0.955 **	0.780 *	0.800 *	−0.300
**DCP Macular**	0.800 *	0.800 *	−0.300	−0.024	−0.345	−0.296
**SCP Peripheral**	0.200	0.200	−0.257	−0.037	−0.164	−0.309
**DCP Peripheral**	0.200	0.200	−0.700 *	0.585 **	0.782 **	−0.351

Significance code: * *p* < 0.05 ** *p* < 0.001.

## Data Availability

The datasets generated during and/or analyzed during the current study will be available from the corresponding author on reasonable request.
